# Construct validity of the self-report instrument of perceived stress in the general Costa Rican population of retirement age: CRELES study

**DOI:** 10.3389/fpubh.2025.1568416

**Published:** 2025-04-11

**Authors:** Ericka Méndez-Chacón

**Affiliations:** Centro Centroamericano de Población y Escuela de Estadística, Universidad de Costa Rica, San José, Costa Rica

**Keywords:** perceived stress, Rasch model, adults of retirement age, validity, reliability

## Abstract

**Background:**

It is known that the effects of stress on the body harm health and mortality outcomes. Applying self-report instruments to the general population can help identify degrees of stress and provide evidence on how stress affects social relationships, health, and even mortality. This research aims to explore the internal validity of questions of perceived stress in the general Costa Rican population close to pension or retirement age.

**Methods:**

A nationally representative sample of 1,469 individuals born between 1945 and 1955 in Costa Rica completed a series of questions related to perceived stress. Factor analysis, elements of classical test theory, and a Rasch model were used to generate evidence of scale validity.

**Results:**

Adequate internal consistency was obtained by factor analysis, with one factor explaining the whole of the variability. The Omega Index value was 0.6261. The fit values (INFIT) detected by the Rasch model range between 0.9 and 1.2.

**Conclusion:**

The items form a scale that refers to the construct of perceived stress and has sufficient internal consistency. However, it is imperative to generate a more substantial number of items to enhance this construct’s precision of measurement.

## 1 Introduction

The term “stress” in living organisms was defined by (60), building upon the knowledge of the time that Bernard and Cannon had already defined about the *milieu intérieur*, which referred to the balance of the internal environment through the homeostasis of the interstitial fluid (1). Selye conceived the organism as a dynamic system seeking to maintain that internal equilibrium, known as homeostasis (2). He defined stress as “a non-specific response of the body to any demand for change” (3–6).

Seyle conceptually distinguished stress from a “stressor,” with the stressor representing any condition or factor that elicits a response. The definition of stress put forth by Selye originated from experimental findings in animals, particularly rats (6, 7), and was subsequently extended to humans.

Since being defined by Selye, stress has been widely studied, and numerous authors have expanded or adapted the concept as research on the subject has deepened. Miller et al. (8), drawing on Cohen et al.’s (9) definition, define stress “as a process involving a stimulus, the evaluation of the stimulus, and a response.”

When stressors, which are stimuli perceived as threatening and difficult to manage, are encountered, they induce a psychological state experienced as stress and trigger a series of behavioral and biological adjustments, known as responses (8, 10).

Physiological reactions to stress involve the integration of responses from the hypothalamic–pituitary–adrenal (HPA) axis, the central nervous system (CNS), the autonomic nervous system (ANS), and the sympathetic nervous system (SNS) (1, 11, 12).

Stress, coping strategies, and emotions are explained by Lazarus and Folkman’s (10) transactional model. According to this model, stress is viewed as the result of the interaction between the individual and their environment. It recognizes that stress is not solely determined by the isolated environmental event or response but rather by the individual’s interpretation of the situation. This perception plays an important role in the emotional and physiological responses to an event (13). This is how the concept of “perceived stress” arises.

In 1995, Cohen emphasizes on the organisms’ perception and assessment of the potential harm posed by objective environmental experiences. When individuals perceive that the demands of their environment surpass their coping capacities, they label themselves as being under stress and subsequently experience a concurrent negative emotional response. In other words, events influence individuals who perceive them as stressful (9).

The effects of stress on the body have been extensively studied. For instance, chronic exposure to stress hormones such as adrenaline and cortisol has been found to impact brain structures involved in cognition and mental health (14). Additionally, stress has been associated with increased serum levels of inflammatory markers such as interleukin-6 (IL-6) and C-reactive protein (CRP) in the bloodstream. (15–18). Moreover, stress has been linked to various health outcomes including diabetes, depression, cardiovascular disease, delayed wound healing, progression of autoimmune conditions, and even mortality (13, 19, 20).

Perceived stress has traditionally been measured using the Perceived Stress Scale (PSS), a psychological tool created by Cohen and colleagues consisting of 10 questions. The PSS aims to measure the extent to which individuals perceive their general life situations as stressful. The initial version of the scale included 14 questions and was evaluated in two samples of college students, as well as in a sample of individuals participating in a community smoking cessation program in Oregon, USA. The Cronbach’s reliability coefficient for each sample was found to be 0.84, 0.85, and 0.86, respectively (21).

Despite being widely used, Taylor’s literature review highlights three significant considerations regarding the validity of the 10-item Perceived Stress Scale (PSS): (a) there is ongoing debate regarding whether a one- or two-factor model better represents the relationship among the scale items, (b) limited information is available regarding the performance of individual items on the scale, and (c) it is unclear whether PSS scores are subject to gender bias (22).

On the other hand, the original 60-item General Health questionnaire (GHQ) was developed by Goldberg and Williams (61)to fulfill two main objectives: (1) to identify the inability to perform regular functions, and (2) to detect the emergence of distressing phenomena. Unlike the PSS, the GHQ primarily focuses on disruptions in normal functioning rather than examining traits throughout the lifespan. It specifically targets personality disorders or adjustment patterns associated with distress, such as depression, anxiety, social disorder, and hypochondriasis. The 12-item version of the GHQ has shown Cronbach’s coefficients ranging from 0.82 to 0.90 (23).

The GHS has been employed in various studies conducted in different cities worldwide, including Ankara, Athens, Bangalore, Berlin, Groningen, Ibadan, Mainz, Manchester, Nagasaki, Paris, Rio de Janeiro, Santiago de Chile, Seattle, Shanghai, Verona, and the United Kingdom. It has also been widespread in the United States (24). Although the scale has been validated, extensively utilized, found to be reliable and unidimensional, and possesses no response bias, recent studies suggest that none of these assumptions may be correct. There are concerns about potential response bias in the negative items, which may limit its utility as a screening tool for psychiatric morbidity, particularly in general population settings (25).

The Health and Living Status of the Elderly in Taiwan study (HLSET),^1^ which examines the social, economic, and physical well-being of the older adult population in Taiwan, employed a set of six questions to measure perceived stress. The questions covered various domains, including (1) health, (2) financial situation, (3) work, (4) relationship with family members, (5) health, financial situation, work, or marriage of a family member, and (6) any other situation (26).

Participants were asked to indicate their level of pressure or anxiety in response to these situations. Their responses were coded using a scale where 0 represented “none,” 1 represented “somewhat,” and 2 represented “much” (26, 27). Cronbach’s alpha for the 1999, 2003, and 2007 waves was 0.68, 0.65, and 0.64, respectively (26).

The utilization of self-report instruments within the general population―i.e., individuals who are not hospitalized or exclusively receiving treatment for any specific pathology―can help identify varying levels of stress and provide insight into the mechanisms through which stress impacts social relations, health, and even mortality. This information is essential for the development of public policies aimed at stress management and ultimately improving the overall health of the population.

The study of stress in individuals approaching retirement is highly significant, as this demographic stands at a crucial point in life, characterized by a major transition. These individuals face the need to adjust to a new daily routine and prepare for the changes that lie ahead. Additionally, this age group is prone to experiencing anxiety and stress due to factors such as economic conditions, shifts in personal relationships, uncertainties about the future, the unavoidable aging process, and ongoing health issues. These factors are considered critical in influencing healthy aging (28–30).

Costa Rica, a Latin American country, boasts a remarkable life expectancy and impressive historical indicators of good health (31–34). Whole population of the country is entitled to social security benefits, and it should be noted that the nation has not maintained an army since 1948. Notably, the Costa Rican region of Nicoya is recognized as a Blue Zone, where its inhabitants exhibit exceptionally favorable health characteristics compared to the rest of the country (33, 35).

The nation’s achievements in health, including the reduction of infant mortality rates, are likely attributed to its political and socio-economic circumstances throughout history. These circumstances have likely contributed to creating a conducive environment for low-stress living, leading to Costa Rica being ranked as the happiest country in the world according to the Happy Planet Index in 2009 (36–38). The circumstances of the country and the life and health characteristics of Costa Ricans, mentioned above, make Costa Rica a good case study to better understand the relationship between stress and health.

As will be explained in the methodology section, this country has data from a longitudinal study at the national level. The CRELES-RC 2010 survey administered this set of five questions to measure perceived stress in a national sample of individuals born between 1945 and 1955. It is important to note that this scale has not been psychometrically evaluated, and to the best of the author’s knowledge, there is no other study at the national level that carries out research of this magnitude. Despite the evident changes in health and social conditions resulting from the pandemic, the evaluation of these questions provides a methodological basis for seeking a more precise measurement of stress in future general population studies. This research aims to research the internal validity of questions on perceived stress among the Costa Rican population close to pension or retirement age.

## 2 Methodology

### 2.1 Participants

The Costa Rica Longevity and Healthy Aging Study (CRELES) is a set of longitudinal, nationally representative surveys of the health and life experiences of older adults in Costa Rica. CRELES is part of a growing set of health and retirement surveys being conducted around the world, including studies led in the United States (39), Mexico (40), and other Latin American countries (41–43).

The CRELES retirement cohort, known as CRELES-RC 2010 contemplates individuals born between 1945 and 1955, with interviews conducted starting in 2010. The sample consists of 2,798 eligible participants at baseline. Data from the study can be accessed at http://creles-download.demog.berkeley.edu/CRdata.pl.

In order to meet the objective of this study, the following were defined as inclusion criteria:

- People born between 1945 and 1955.
- People resident in Costa Rica at the time of the study.
- People who completed all the questions related to stress.
- People who answered the questionnaire by their own means.

The following individuals were excluded:

- People who required an assistant (proxy) to answer the questionnaire.
- Individuals who did not respond to a question related to stress measurement because it did not apply to them were also excluded, for example, those not currently employed were not asked if they felt stressed or anxious about their work.

The present study utilized a sample of 1,469 individuals who had responded to all the stress-related questions.

CRELES-RC was conducted by the Centro Centroamericano de Población (CCP) at the University of Costa Rica in collaboration with the University of California, Berkeley. Funding for the study was provided by the U.S. National Institute on Aging (NIH R01 AG031716).

### 2.2 Stress measurement tools

Perceived stress was measured by the following series of questions:

- Problems at your **job**, do they make you feel stressed or anxious?
- **Family relationships**, do they make you feel stressed or anxious?
- Your **health**, does it make you feel stressed or anxious?
- Your **financial situation**, does it make you feel stressed or anxious?
- **Parents or other family members’ health**, does it make you feel stressed or anxious?

These questions are very similar to the questions used in The Health and Living Status of the Elderly in Taiwan study (HLSET) (see text footnote 1) (26). In the aforementioned study, the response options to each question were as follows: 0 = ‘none’, 1 = ‘somewhat’, and 2 = ‘much’. In the case of Costa Rica, however, the response options were limited to a binary “yes” or “no” choice.

Data from 1,469 individuals were analyzed, for including only the cases where all items of the scale were fully completed. If an individual required another person (proxy) to answer the interview, stress-related questions were not asked, and those cases were excluded from this analysis. The response options were yes or no.

### 2.3 Data analysis

The first step was to validate whether the five questions were aligned in the same direction to form a scale. This is known as the unidimensionality of the questions. The factor analysis of principal components (FAPC) was applied, using a tetrachoric correlation matrix (44). Factor analysis is a multivariate technique commonly used in fields such as psychology or other behavioral and health disciplines. It is employed when measuring certain concepts directly results challenging, as in this case, stress.

The current practice is to measure concepts indirectly by collecting information on related aspects that can be directly measured or observed. These related aspects are aggregated into a single construct, known as the “latent variable,” which is assumed to be an indicator of the concept being measured. Hence, factor analysis helps establish the relationship between the latent variable (not directly measured) and the observed variables (approximated).

The underlying model of the method is essentially multiple linear regression where the focus of interest is on explaining the covariance or correlation structure (or both), this helps determine whether the p response variables exhibit patterns of relationship among themselves, allowing them to be defined in m subsets of closely related variables that differ from the variables in other subsets.

In addition, Cronbach’s alpha (45) and Omega (46) coefficients were calculated as reliability coefficients. A maximum value of 1 of Cronbach’s alpha coefficient indicates a maximum level of reliability (45, 47), meaning the observed responses are reproducible. Factor analysis and calculation of coefficients were performed using Stata.

Another evaluated aspect was the presence of items that are either difficult to answer or do not contribute effectively to the measurement of perceived stress. A Rasch analysis was used for this purpose. According to Prieto and Delgado (48), Rasch analysis is based on a mathematical model that describes the relationship between the probability of a correct (or predefined) response to an item and the difference between the ability of the respondent. The procedure assesses the fit of each response and item to a unidimensional model, where a single construct or latent variable underlies and is reflected in the correct response to the item (49). In the context of knowledge tests, the Rasch model posits that the probability of a given response being obtained is contingent on the respondent’s level of knowledge regarding the construct being measured, as well as the difficulty of the item. In the affective domain, the term ‘difficulty’ is employed to denote the extent to which the mean score on a specific item deviates from the mean overall. In this context, it is also referred to as endorsability (50). The Rasch model analysis was performed in the Winsteps package, version 3.80.1.

## 3 Results

### 3.1 Description

Table 1 shows the main sociodemographic characteristics of the participants. The study included a total of 641 women and 828 men, 5% of whom were living in the blue zone of Nicoya. The average age was 59 years for both groups. Among women, 57% reported being married or in a consensual union, while this percentage rose to 84% among men. Furthermore, nearly 6 out of 10 participants indicated having completed only elementary or basic education high school, with 56% of women and 57% of men falling into this category.

**Table 1:** Socioeconomic characteristics (averages or proportions) by sex.

Variables (*n*)	Units	Females (*n* = 641)			Males (*n* = 828)		
		Mean	LI	LS	Mean	LI	LS
**Demographics and socioeconomics**							
Nicoya region	Binary 0–1	0.05	0.02	0.11	0.05	0.03	0.10
Age	Years	59.01	58.74	59.28	59.12	58.84	59.41
Marital status	Binary 0–1						
Married or civil union		0.57	0.52	0.62	0.84	0.81	0.87
Other		0.43	0.38	0.48	0.16	0.13	0.19
Level of education	Binary 0–1						
Elementary		0.56	0.49	0.63	0.57	0.51	0.63
Secondary and further		0.44	0.37	0.51	0.43	0.37	0.49

Estimates include corrections for complex sampling design and weighing factors.

**Figure 1:**
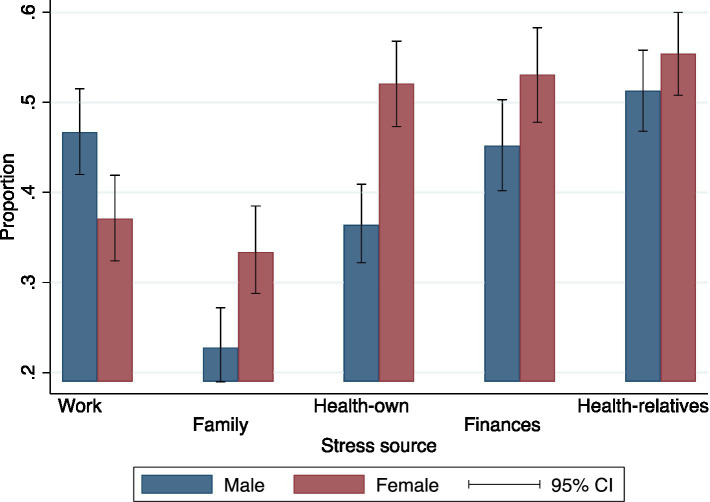
Proportion of individuals who feel stressed, by source of stress and sex. Estimates include corrections for complex sampling design and weighing factors.

The highest prevalence of stress was attributed to one’s health, financial situation, and parental health. Moreover, there were statistically significant differences in the reporting of stress causes between men and women. For instance, 36% of men and 52% of women reported stress related to their health. This pattern of higher prevalence among women was consistent for all other stress causes, except for work-related stress (Figure 1).

### 3.2 Reliability of the questions as a scale

#### 3.2.1 Unidimensionality

The tetrachoric correlations between the items show a medium-intensity relationship between them, as the correlations are of the order of 0.27 or higher between items (Table 2). The present study demonstrates a robust correlation between perceived stress due to financial issues and stress due to one’s own health (*r* = 0.61). Furthermore, a significant correlation is also demonstrated between perceived stress due to finances and stress due to work (*r* = 0.52).

**Table 2:** Tetrachoric correlation matrix.

Item	Health-own	Finances	Work	Family	Health-relatives
Health-own	1				
Finances	0.608	1			
Work	0.395	0.517	1		
Family	0.444	0.364	0.291	1	
Health-relatives	0.315	0.272	0.331	0.305	1

**Figure 2:**
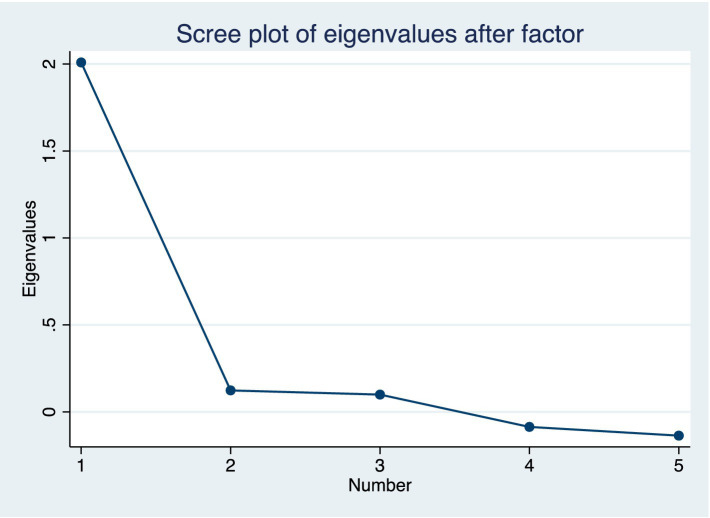
Cattell’s criterion for selecting factors.

**Figure 3:**
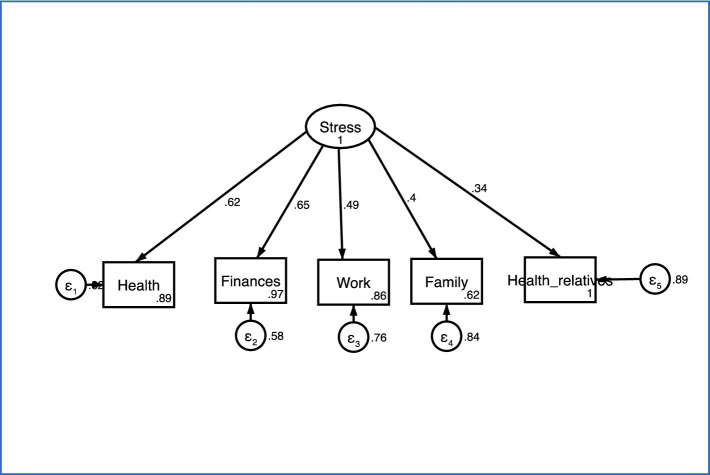
Confirmatory factor model (standardized estimations). RMSEA = 0.061; CFI = 0.964; TLI = 0.928.

An exploratory factor analysis (by principal components) was performed, using this structure of tetrachoric correlations and the result revealed the presence of a single dimension. According to Cattell’s criterion, the items form a single factor (Figure 2). This factor explains 100% of the total variance. The Kaiser-Meyer-Olkin test yielded a value of 0.757, suggesting that data fit the factor analysis and supporting the hypothesis that data are correlated and that, in this case, a single coherent or latent factor can be identified (Table 3). Confirmatory factor analysis is a statistical technique used to verify the proposed structure of a measurement model. It provides evidence that only one construct is being measured, and that the model fits the data well (Figure 3).

**Table 3:** Exploratory factor analysis.

Factor	Eigenvalue	Difference	Proportion	Cumulative
Factor1	2.01	1.89	1.00	1.00
Factor2	0.12	0.02	0.06	1.06
Factor3	0.10	0.19	0.05	1.11
Factor4	−0.09	0.05	−0.04	1.07
Factor5	−0.14	.	−0.07	1.00
LR test: independent vs. saturated: chi2(10) = 1,795.69 Prob>chi2 = 0.0000				

Factor loadings (pattern matrix) and unique variances		
Variable	Factor1	Uniqueness
Health-own	0.7512	0.4357
Finances	0.7644	0.4157
Work	0.6084	0.6299
Family	0.5384	0.7102
Health-relatives	0.4481	0.7992

Factor analysis/correlation (Number of obs = 1,469).

Method: iterated principal (Factors retained factors = 1).

Rotation: (unrotated) (Number of params = 5).

#### 3.2.2 Reliability

Based on the results of the factor analysis confirming the unidimensionality of the questions, a reliability analysis was conducted using classical test theory indicators, including the Alpha and Omega coefficients, as well as a Rasch model. Table 4 shows the statistics of the items under study. Indicators, such as Cronbach’s alpha and the Omega coefficient, measure the internal consistency of the items which, in general terms, is an evaluation of how reliably they measure the construct they are intended to approximate. In this study, these indicators yielded values of 0.6215 and 0.6261, respectively.

**Table 4:** Item statistics (*n* = 1,469).

Stress source	Mean	CI95%	
Own health	0.42	0.38	0.45
Financial situation	0.48	0.44	0.52
Work problems	0.43	0.40	0.47
Family relationships	0.26	0.23	0.30
Relatives health	0.53	0.49	0.56
Alpha’s Cronbach	0.6215		
Omega’s coefficient	0.6261		

##### 3.2.2.1 Consistencia interna

Table 5 shows the correlation values for each item as well as Cronbach’s alpha. The items show good discrimination indexes. The issue of low indicator values arises from the limited number of items considered for the measurement of this construct.

**Table 5:** Reliability statistics.

Item	Obs	Sign	Item-test correlation	Item-rest correlation	Inter-item covariance	Alpha
Own health	1,469	+	0.686	0.447	0.052	0.530
Financial situation	1,469	+	0.690	0.451	0.051	0.527
Work problems	1,469	+	0.637	0.378	0.058	0.566
Family relationships	1,469	+	0.568	0.319	0.066	0.594
Relatives health	1,469	+	0.569	0.285	0.067	0.613
Test scale					0.059	**0.622**

Bold value 0.622 represents Alpha’s Cronbach.

#### 3.2.3 Rasch model

The Rasch model allows solving some of the deficiencies of the Classical Test Theory, particularly, joint measurement, by expressing parameters of individuals and items in the same units. It also allows the quantification of the fit of the response patterns to the model, as responses to the items solely depend on the individual’s skill levels, or in this case, on the perception of stress.

The reliability of the items indicates the consistency of the estimates of the difficulty parameter if the same set of items is applied to another group of individuals with similar characteristics. In this case, the estimated value was 1.00, indicating that the Rasch estimates are highly consistent. This result can be attributed to the large sample size. It’s worth noting that while this indicator is similar to Cronbach’s alpha in the Classical Theory approach, they are not comparable.

The main objective of the Rasch model is to identify two aspects: (1) people who answer the items positively because they really are under conditions that generate stress and (2) properly formulated items that are only answered positively by people who really feel stress. This is referred to as model fit.

To assess individuals’ fit to the model, difficulty measures and fit statistics (INFIT MNSQ) were employed. According to the measure indicator, the item “Relatives health” was found to be the easiest (more frequent), with a higher proportion of individuals reporting stress related to the health of their parents or family members (53%) compared to other items. Conversely, “Family relationships” was the most difficult item (less frequent); 26% of individuals reported feeling stressed about this activity. The INFIT values for all five items fell within the acceptable range of 0.9–1.2, indicating adequate fit (Table 6) (48). During the study, differential item functioning (DIF) was also assessed. This assessment’s results showed significant differences in items related to perceived stress due to one’s health, relationships with family, and work. In contrast, no differences were found in stress-related items due to the financial situation and the health of parents or close relatives.

**Table 6:** Item statistics: measure order.

Entry number	Total score	Total count	Measure	Model	Infit		Outfit		PTMEA	Exact	Match	Item
				S.E.	MNSQ	ZSTD	MNSQ	ZSTD	CORR.	OBS%	EXP%	
4	406	1,469	1.1	0.08	1.07	1.9	1.12	1.9	0.57	75.1	77	Family
3	624	1,469	0	0.07	0.99	−0.2	0.99	−0.3	0.63	70.1	70.6	Work
1	648	1,469	−0.11	0.07	0.89	−4.1	0.87	−3.7	0.68	75.8	70.4	Health-own
2	714	1,469	−0.42	0.07	0.89	−4.2	0.86	−3.9	0.68	73.6	69.2	Finances
5	748	1,469	−0.57	0.07	1.16	5.6	1.19	4.7	0.58	63.1	70	Health-relatives
Mean	514	1,091	0	0.07	1	−0.2	1.01	−0.3		71.5	71.4	
S.D.	119.6	0	0.59	0	0.11	3.7	0.13	3.3		4.7	2.8	

**Figure 4:**
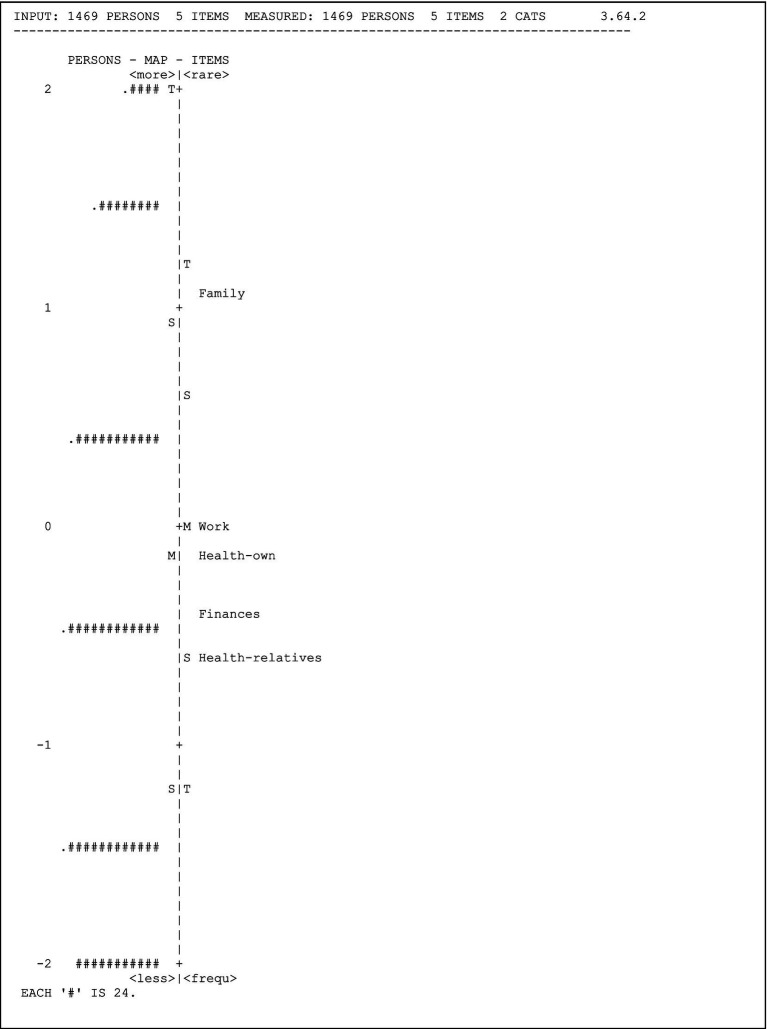
Person-item map.

Figure 4 presents the person-item map, which illustrates the joint distribution of individuals’ measures and item locations on the same measurement continuum. Individuals are depicted on the left side, while items are on the right. The map should ideally show items located at various points along the scale, indicating significant differences in measurement. Individuals who responded positively to more difficult items or situations are positioned at the top, while those who responded positively to easier items are located at the bottom. In this case, although the map adequately depicts the distribution, it is evident that additional items need to be added to the instrument, to ensure that they measure the same construct.

Finally, Table 7 represents a resume that compares results between the test classical theory and the modern techniques. Information is also presented on people’s adjustment to the model, which is quite acceptable according to the measurement indicators. It is important to acknowledge that, given its nature as a social construct, individuals may experience varying degrees of stress across various domains.

**Table 7:** Information table about items’ properties.

Classic test theory		Rasch model		
Item	Item-test correlation	Item	MNSQ	Measure
Health-own	0.69	Health-own	0.89	−0.11
Finances	0.69	Finances	0.89	−0.42
Work	0.64	Work	0.99	0
Family	0.57	Family	1.07	1.1
Health-relatives	0.57	Health-relatives	1.16	−0.57
Alpha’s Cronbach	0.62	Mean	1	0
Omega’s coefficient	0.63	S.D.	0.11	0.59
Cattell’s criterion	One factor	Person information		
		Mean	1.00	−0.40
Origin	The Healthy Aging Longitudinal Study in Taiwan (HALST)	Median	1.00	−0.45
		Min	0.60	−2.88
		Max	1.59	2.92
		SD	0.22	1.69

## 4 Discussion

This study reveals that stress is most reported concerning personal health, financial circumstances, and the health of parents and relatives. Furthermore, it was found that there are significant differences in the perception of stress between men and women, with women reporting higher levels of stress. Data show that women in Costa Rica report higher stress levels than men (51), similar to women in U.S. populations (52). This phenomenon is widely understood (10), and some authors describe it as connected to the context and the social construction of ideas, beliefs, and the cultural, economic, and political aspects cultures create regarding gender roles and stereotypes (53).

In terms of the measurement of perceived stress using the items employed in this study, the Classical Test Theory (TCT) analysis indicates that the scale demonstrates good internal consistency. The factor analysis supports the unidimensionality of the construct, with all items loading onto a single dimension and explaining a substantial proportion of the variability in the data. The reliability coefficients, such as Cronbach’s alpha and Omega, were found to be 0.6215 and 0.6261, respectively, when considering binary response data.

The result of the application of the Rasch model demonstrates an adequate overall fit of the items to the model. However, it is worth noting that the item related to stress about the health of parents or relatives shows some lack of fit, as it reflects the highest prevalence of stress. In conclusion, the items included in the scale exhibit sufficient internal consistency and collectively represent the construct of perceived stress. In the context of DIF, it is essential to recognize that men and women show different responses. However, it is suggested that this phenomenon should not result in the invalidation of the items, as there are theoretical justifications for the existence of gender-related differences (53).

The items utilized in this research are also applied in the Taiwan Longitudinal Study on Aging (TLSA) (54). Both the Taiwan and Costa Rica studies are part of the growing set of health and retirement surveys (HRS) being carried out in other latitudes, such as the United States, (39), Mexico (40) and other Latin American countries (41–43). These studies were designed to be comparable with each other and do not use specific scales to measure stress such as the Stress Appraisal Measure (SAM) (55), Impact of Event Scale (IES) (56) or the Perceived Stress Scale (PSS) (21). However, recent applications of this type of survey have seen researchers trying to harmonize the measurement of these constructs.^2^ Cronbach’s alpha observed for Costa Rica is lower than that observed in Taiwan. Although these are low reliability values, the factors can be attributed to the small number of items included and to the fact that, unlike Taiwan, the measurement in Costa Rica was done based on a binary scale (yes–no).

There have been no population studies in Taiwan that have reported using the Rasch analysis. For its part, this study offers a broader perspective as it further explores the construct validity and dimensionality of the stress measurement questions by applying a modern theory within the framework of parametric analysis, as Rasch models do.

The use of the Rasch model in this study is a significant strength as it overcomes the limitations of the Classical Test Theory. Additionally, the study benefits from a large sample size and the random selection of a general population, rather than focusing solely on hospitalized individuals. However, it is important to acknowledge that the items used in this study have a disadvantage related to their wording. They may appear to be asking about two situations or emotions simultaneously when assessing whether the situation causes stress or anxiety. Moreover, the limited quantity of items is a significant constraint in this context.

Perceived stress is commonly described in the literature as the extent to which an individual assesses their life as stressful. It encompasses emotions such as feeling stressed, upset, or angry, as well as cognitive evaluations that one lacks control or that the demands of a situation outweigh the available coping resources (57). On the other hand, anxiety is described as an anticipated emotional response to perceived danger or threat, which can occur independently of specific stressors (58, 59) and is often associated with uncertainty about the future. Therefore, it is recommended that future research treat these concepts separately and explore them individually.

Based on the psychometric indicators, the items used in this study do form a scale for measuring perceived stress. However, it is recommended to expand the scale by including a greater number of items that clearly measure the same construct. This would help improve the statistical properties of the scale and provide a more robust instrument for research purposes and the development of policies aimed at enhancing health and quality of life.

For future studies, it is suggested to compare the scale used in this study with established stress measurement scales such as the Perceived Stress Scale (PSS). It is also recommended to complement the scale with an objective measurement such as cortisol to serve as a gold standard for validating the items presented in this study. This could also lead to the development of new items that provide a more accurate assessment of perceived stress.

In addition, to ensure an understanding of the items and the measurement of the construct, it is advisable to conduct qualitative interviews as a complementary approach. Furthermore, it would be desirable for the questions to be applicable to all populations, including individuals who do not work outside the home. It is important to note that the sample size for this study was reduced due to the fact that the items were not applied to all participants. This is a limitation of the study.

In conclusion, the questions used in this study are appropriate for measuring stress. The findings provide statistical evidence for a unidimensional scale, as supported by factor analysis and the Rasch model, which is a recognized standard for modern psychometric assessments of outcome scales. To enhance the scale’s reliability and validity, it is recommended to expand the number of items, ensuring they measure the same construct of perceived stress. Additionally, comparing the results with an objective measurement such as cortisol can provide further validation and a more comprehensive understanding of stress levels.

## Data Availability

The datasets presented in this study can be found in online repositories. The names of the repository/repositories and accession number(s) can be found at: http://creles-download.demog.berkeley.edu/CRdata.pl.

## Footnotes

^1^ https://adcnet.isr.umich.edu/survey/health-and-living-status-of-the-elderly-in-taiwan-hlset/

^2^ https://www.stressmeasurement.org/hrs-harmonization-project
